# Cross-sectional associations between sleep duration, sedentary time, physical activity, and adiposity indicators among Canadian preschool-aged children using compositional analyses

**DOI:** 10.1186/s12889-017-4852-0

**Published:** 2017-11-20

**Authors:** Valerie Carson, Mark S. Tremblay, Sebastien F. M. Chastin

**Affiliations:** 1 Faculty of Physical Education and Recreation, University of Alberta, Edmonton, AB T6G 2H9 Canada; 2 Healthy Active Living and Obesity Research Group, Children’s Hospital of Eastern Ontario Research Institute, Ottawa, ON K1H 8L1 Canada; 3 Institute for Applied Health Research, School of Health and Social Care, Glasgow Caledonian University, Glasgow, G4 0BA UK

**Keywords:** Sleep, Sedentary behaviour, Physical activity, Adiposity, Preschool children

## Abstract

**Background:**

Sleep duration, sedentary behaviour, and physical activity are three co-dependent behaviours that fall on the movement/non-movement intensity continuum. Compositional data analyses provide an appropriate method for analyzing the association between co-dependent movement behaviour data and health indicators. The objectives of this study were to examine: (1) the combined associations of the composition of time spent in sleep, sedentary behaviour, light-intensity physical activity (LPA), and moderate- to vigorous-intensity physical activity (MVPA) with adiposity indicators; and (2) the association of the time spent in sleep, sedentary behaviour, LPA, or MVPA with adiposity indicators relative to the time spent in the other behaviours in a representative sample of Canadian preschool-aged children.

**Methods:**

Participants were 552 children aged 3 to 4 years from cycles 2 and 3 of the Canadian Health Measures Survey. Sedentary time, LPA, and MVPA were measured with Actical accelerometers (Philips Respironics, Bend, OR USA), and sleep duration was parental reported. Adiposity indicators included waist circumference (WC) and body mass index (BMI) z-scores based on World Health Organization growth standards. Compositional data analyses were used to examine the cross-sectional associations.

**Results:**

The composition of movement behaviours was significantly associated with BMI z-scores (*p* = 0.006) but not with WC (*p* = 0.718). Further, the time spent in sleep (BMI z-score: *γ*_*sleep*_ = −0.72; *p* = 0.138; WC: *γ*_*sleep*_ = −1.95; *p* = 0.285), sedentary behaviour (BMI z-score: *γ*_*SB*_ = 0.19; *p* = 0.624; WC: *γ*_*SB*_ = 0.87; *p* = 0.614), LPA (BMI z-score: *γ*_*LPA*_ = 0.62; *p* = 0.213, WC: *γ*_*LPA*_ = 0.23; *p* = 0.902), or MVPA (BMI z-score: *γ*_*MVPA*_ = −0.09; *p* = 0.733, WC: *γ*_*MVPA*_ = 0.08; *p* = 0.288) relative to the other behaviours was not significantly associated with the adiposity indicators.

**Conclusions:**

This study is the first to use compositional analyses when examining associations of co-dependent sleep duration, sedentary time, and physical activity behaviours with adiposity indicators in preschool-aged children. The overall composition of movement behaviours appears important for healthy BMI z-scores in preschool-aged children. Future research is needed to determine the optimal movement behaviour composition that should be promoted in this age group.

## Background

Sleep, sedentary behaviour, and physical activity are three behaviours that are distributed throughout a 24-h period that fall on the movement/non-movement intensity continuum. These three behaviours have recently been referred to collectively as “movement behaviours” [1]. The study of the health implications of movement behaviours is a growing research area [1–5] that is gaining increased public health interest [6]. Traditionally, research has studied the association between individual movement behaviours and health separately, and this evidence has subsequently informed separate and distinct recommendations for adequate sleep, minimal sedentary behaviours, and regular physical activity [7–10]. However, the recent development and release of *Canadian 24-Hour Movement Guidelines for Children and Youth* (5 to 17 years) presents a paradigm shift in how we conceptualize and promote integrated movement behaviours combinations for optimal health [1].

Increasing and strengthening the evidence base with regard to the associations between integrated 24-h movement behaviours and health has its challenges. One challenge is accurately measuring movement behaviours within a 24-h period in free-living settings [2]. Recent research suggests there is no single monitoring device that can accurately capture all movement behaviours in the field [2]; however, technology continues to evolve and advance at a rapid pace in this area. Another challenge is utilizing appropriate analytical methods to examine the associations between movement behaviours and health. More specifically, due to the co-dependent nature of sleep, sedentary time, and physical activity data, traditional statistical techniques (e.g., regression) should not be used because of collinearity issues that arise when the time spent in all movement behaviours is included in the same model [4]. Compositional analysis has recently been introduced as one appropriate method for analyzing movement behaviour data, to deal with issues of collinearity and the intrinsic compositional nature of these data [4, 11]. Unlike traditional statistical techniques, which use open space (i.e., orthonormal) geometry where continuous numerical data can vary from negative infinity to positive infinity, compositional analyses specifically deal with co-dependent data by using closed space (i.e., simplex) geometry where data are constrained (e.g., 24-h period) [4]. This eliminates collinearity problems and deals with the co-dependence between time spent in different movement behaviours.

Recent research utilizing compositional analyses in Canadian school-aged children and youth [3] and American adults [4] has shown consistent findings for adiposity indicators (i.e., body mass index (BMI), BMI z-score, waist circumference). Specifically, the *composition of movement behaviours* was significantly associated with adiposity indicators in both samples [3, 4]. Furthermore, relative to the time spent in the other movement behaviours, moderate- to vigorous-intensity physical activity (MVPA) and sleep tended to be negatively associated, and sedentary behaviour and light-intensity physical activity (LPA) tended to be positively associated, with adiposity indicators in both samples [3, 4].

To our knowledge no study has used compositional analyses to examine the associations between movement behaviours and adiposity indicators in preschool-aged children (3 to 4 years). These children are not immune to current public health concerns around inactivity, sleep deprivation, and obesity. For instance, 42 million children <5 years worldwide are classified as overweight or obese [12], and a large proportion (≥85%) of preschool-aged children are not meeting physical activity and sedentary behaviour guidelines [13, 14]. Furthermore, childhood sleep duration has been declining over time [15].

Early intervention of movement behaviours has recently been identified as a promising strategy for obesity prevention, given that behavioural patterns and obesity status in early childhood have been shown to track over time [16]. Consequently, an understanding of the associations between movement behaviours and adiposity indicators from an integrated perspective in this age group is important for informing future interventions to help prevent obesity. Therefore, the objectives of this study were to examine: 1) the combined associations of the composition of time spent in sleep, sedentary behaviour, LPA, and MVPA, with adiposity indicators; and 2) the association of the time spent in sleep, sedentary behaviour, LPA, or MVPA with adiposity indicators relative to the time spent in the other movement behaviours in a representative sample of Canadian preschool-aged children.

## Methods

### Participants

Participants were children aged 3 to 4 years from cycles 2 (2009–2011) and 3 (2012–2013) of the Canadian Health Measures Survey (CHMS) [17]. Both cycles collected cross-sectional data from a nationally representative sample of 3- to 79-year-olds living in private households. Data collection entailed an interview in the participant’s home followed by a visit to a mobile examination centre for a physical health examination. At the end of the physical health examination, participants were given an accelerometer to wear during waking hours for 7 consecutive days. A total of 789 participants aged 3 to 4 years across cycles 2 and 3 were eligible for this study. Ethics approval for the CHMS was obtained from Health Canada and the Public Health Agency of Canada Research Ethics Board [18]. Written informed consent was obtained from a parent or guardian, and assent was obtained from the child [19]. Detailed information about the CHMS is available elsewhere [19–21].

### Movement behaviours

Sedentary time, LPA, and MVPA were objectively measured with Actical accelerometers (Philips Respironics, Bend, OR, USA). Participants wore the accelerometers on an elastic belt around their waist over their right hip for 7 consecutive days. Data were collected in 60-s epochs in cycle 2 and 15-s epochs in cycle 3. The memory capacity of the Actical accelerometers used in the CHMS allowed for 7 days of data to be recorded for each participant in cycle 2 but for only 5.6 days for cycle 3 participants. In order for data to be comparable across both cycles, days 6 and 7 were excluded for participants in cycle 2. Since the physical health examinations occurred 7 days a week, days 6 and 7 for participants varied across the week. Non-wear time in cycle 2 was defined as ≥60 consecutive minutes of zero counts, with allowance for 2 min of counts between zero and 100. Non-wear time in cycle 3 was defined as ≥240 intervals of 15 s of zero counts, with allowance for 30 s of counts between 0 and 25 [22, 23]. In both cycles, a valid day was defined as ≥5 h of wear time [23]. To be included in the analyses, participants were required to have 3 or more valid days [24, 25]. In cycle 2, sedentary time was defined as <100 counts per minute (cpm) [26], LPA as 100–1149 cpm, and MVPA as ≥1150 cpm [27]. In cycle 3, sedentary time was defined as <25 counts per 15 s, LPA as 25–278 counts per 15 s, and MVPA as ≥288 counts per 15 s. The 15-s epoch data of cycle 3 was then converted into minutes/day by dividing the values by 4. Average minutes per day of sedentary time, LPA, and MVPA across valid days were calculated. In order to ensure the data were comparable across cycles, correction factors were used on the minutes/day variables from cycle 2 [28].

Since accelerometers were worn only during waking hours, no objective measure of sleep was available within the CHMS. Sleep was measured by parental report as part of the interview that was administered in participants’ homes. Parents/guardians were asked: “How many hours does your child usually spend sleeping in a 24-hour period, excluding time spent resting?” Responses were recorded to the nearest half hour. Minutes of sleep was calculated.

Average or typical time spent in each movement behaviour (sleep, sedentary behaviour, LPA, MVPA) in a 24-h period were summed. Since both objective and subjective measures were used and sleep was recorded to the nearest half hour, the total time spent in all four movement behaviours did not necessarily equal 24 h (1440 min). Consequently, time spent in movement behaviours was normalized to proportion of the total time [4].

### Adiposity indicators

The adiposity indicators in this study were BMI z-scores and waist circumference. Following Canadian Physical Activity, Fitness and Lifestyle Approach 3rd edition protocols [29], height was measured using a ProScale M150 digital stadiometer (Accurate Technology Inc., Fletcher, NC, USA) and weight was measured using a Mettler Toledo VLC with Panther Plus terminal scale (Mettler Toledo Canada, Mississauga, ON, Canada) by trained health measures specialists. BMI was calculated by dividing weight in kilograms by height in meters squared, and age- and sex-specific BMI z-scores were calculated according to World Health Organization (WHO) growth standards [30]. Following National Institutes of Health protocols, waist circumference was measured at the level of the iliac crest by trained health measures specialists [31].

### Covariates

Similar to previous research on sedentary behaviour, physical activity, sleep, and health in children of the early years [32–34] and based on availability of measures within the CHMS, covariates included age (years), sex (male/female), and highest household education. Highest household education was recoded into 10 categories ranging from “Grade 8 or lower” to “university degree or certificate above bachelor’s degree”. For descriptive purposes, highest household education was categorized and presented in four categories ranging from “less than secondary school” to “post-secondary graduation”.

### Statistical analyses

All statistical analyses were performed using SAS version 9.3 (SAS Institute, Cary, NC, USA). All analyses followed a previously published guide on compositional data analysis for movement behaviours [4] and are consistent with analyses conducted in school-aged children and youth from the CHMS [3]. First, traditional descriptive statistics (i.e., means, standard errors, and frequencies) were calculated for participant characteristics and compositional descriptive statistics were calculated for movement behaviours (i.e., compositional geometric means for central tendency and a variation matrix for dispersion). Values closer to zero in the variance matrix denoted higher co-dependence between movement behaviours, and values closer to 1 denoted lower co-dependence. Compositional geometric mean bar plots were also generated as part of the descriptive statistics to display the relative movement behaviour profiles for the adiposity indicators. For BMI z-score, sub-groups included underweight (<−2.0 standard deviations), normal weight (−2 to 1 standard deviations), at risk for overweight (>1 to 2 standard deviations), and overweight/obese (>2 standard deviations) based on WHO growth standards [30]. Underweight and normal weight sub-groups had to be collapsed due to frequency distributions. For waist circumference, quartiles were used, with quartile one representing low waist circumference and quartile four representing high waist circumference. Further details on the calculations of these descriptive statistics have been previously published [3, 4].

To address objective one of this study, four compositional linear regression models were conducted to examine the association with each adiposity indicator by sequentially rotating the sequence of sleep duration, sedentary time, LPA, and MVPA and entering the composition of movement behaviours into the model via an isometric log-ratio transformation. Model *p*-values were used to determine whether there was a statistically significant association between the composition of movement behaviours and each adiposity indicator. R^2^ coefficients were used to determine the proportion of the variance explained by the composition. Model *p*-values and R^2^ coefficients were identical across all four linear regression models.

To address objective two, the first coefficient and its *p*-value for each rotated model were used to determine whether the individual movement behaviour was significantly associated (positively or negatively) with each adiposity indicator relative to the time spent in the other movement behaviours. All regression models were adjusted for age, sex, and highest household education. Further details on the calculations of these linear regression models have been previously published [3, 4].

Survey weights for combined cycles 2 and 3 accelerometer data, which accounted for non-response and incomplete accelerometer data, were used in all analyses to ensure the sample was representative of Canadian preschool-aged children. To account for survey design effects, the bootstrap technique was used to estimate standard errors and coefficients of variation [35, 36], using 24 degrees of freedom as outlined by the CHMS data user guide [20]. Statistical significance was set a priori at *p* < 0.05.

## Results

Of the eligible 789 participants, 552 had complete data and were included in all analyses. Weighted participant characteristics are presented in Table 1. The average age of the sample was 48.6 months or 3.5 years, and there was a relatively even split between males and females (49.2% female). The geometric means for the % of time spent in each movement behaviour in the 24-h period, and the minutes/day for each movement behaviour after normalizing them to 1440 min/day, are presented in Table 2. On average, participants spent 30.9% of the 24-h period in sedentary time, 15.9% in LPA, 4.5% in MVPA, and 48.7% in sleep. The pair-wise log-ratio variation matrix is presented in Table 3. The two variables with the highest co-dependence were sedentary time and sleep duration (0.0002) and the two variables with the lowest co-dependence were sedentary time and MVPA (0.0012).

**Table 1:** Weighted participant characteristics of the 2009/11, 2012/13 CHMS

Variables	Total (*n* = 552)
Age (years)	3.5 (0.03)
Age (months)	48.6 (0.5)
Sex (%)	
Male	50.8
Female	49.2
Highest household education (%)	
Less than secondary school graduation	5.3^E^
Secondary school graduation	8.1^E^
Some post-secondary	4.8^E^
Post-secondary graduation	81.8^E^
Health indicators	
BMI z-score	0.6 (0.1)
Waist circumference (cm)	50.6 (0.3)

*BMI* body mass index, *CHMS* Canadian Health Measures Survey

Data presented as mean (standard error) for continuous variables and percentage for categorical variables

^E^ = interpret with caution (coefficient of variation 16.6% to 33.3%)

**Table 2:** Geometric Means for SB, LPA, MVPA, and sleep duration in minutes/day (*n* = 552)

Movement behaviour	Minutes/day	% of 24-h
SB	444.5	30.9
LPA	229.5	15.9
MVPA	64.3	4.5
Sleep duration	701.7	48.7

*LPA* light-intensity physical activity, *MVPA* moderate- to vigorous-intensity physical activity, *SB* sedentary time

Movement behaviours have been normalized to 1440 min

**Table 3:** Pair-wise log-ratio variation matrix for SB, LPA, MVPA, and sleep duration in minutes/day (*n* = 552)

	SB	LPA	MVPA	Sleep duration
SB	0	0.0005	0.0012	0.0002
LPA	0.0005	0	0.0006	0.0003
MVPA	0.0012	0.0006	0	0.0008
Sleep duration	0.0002	0.0003	0.0008	0

*LPA* light-intensity physical activity, *MVPA* moderate- to vigorous-intensity physical activity, *SB* sedentary time

**Fig. 1:**
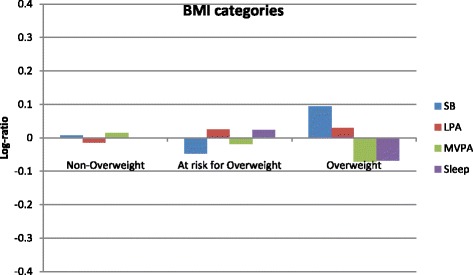
Compositional geometric mean bar plots comparing the compositional mean of the entire sample with the compositional mean of non-overweight, at risk for overweight, and overweight subgroups for sedentary time (SB), light-intensity physical activity (LPA), moderate- to vigorous-intensity physical activity (MVPA), and sleep duration. BMI = body mass index

**Fig. 2:**
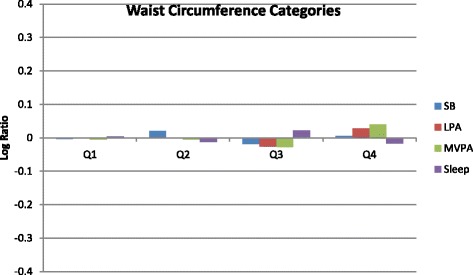
Compositional geometric mean bar plots comparing the compositional mean of the entire sample with the compositional mean of waist circumference quartiles (Q) subgroups for sedentary time (SB), light-intensity physical activity (LPA), moderate- to vigorous-intensity physical activity (MVPA), and sleep duration

Compositional mean bar plots for BMI z-score and waist circumference are presented in Figs. 1 and 2, respectively. For BMI z-score, the greatest log ratios were seen in the overweight group for three of the movement behaviours. More specifically, the proportion of time spent in sedentary behaviour was slightly higher and the proportion of time spent in MVPA and sleep was slightly lower compared to the entire sample. For waist circumference, small log ratios and no clear patterns were observed.

The model *p*-values presented in Table 4 represent the combined associations of the composition of sedentary time, LPA, MVPA, and sleep duration with each adiposity indicator. The composition of movement behaviours was significantly associated with BMI z-score (*p* = 0.006) but not with waist circumference (*p* = 0.718). The model R^2^ indicates that composition of movement behaviours explained 8% of the variance in BMI z-score and 2% of the variance in waist circumference. The association of the time spent in sleep, sedentary behaviour, LPA, or MVPA with each adiposity indicator relative to the time spent in the other movement behaviours is also presented in Table 4. No significant associations were observed.

**Table 4:** Compositional behaviour model for adiposity indicators

Adiposity indicator	Model *p*-value	Model R^2^	γ_SB_	*p*-value	γ_LPA_	*p*-value	γ_MVPA_	*p*-value	γ_sleep duration_	*p*-value
BMI z-score	**0.006**	0.08	0.19	0.624	0.62	0.213	−0.09	0.733	−0.72	0.138
Waist circumference	0.718	0.02	0.87	0.614	0.23	0.902	0.08	0.288	−1.95	0.285

*LPA* light-intensity physical activity, *MVPA* moderate- to vigorous-intensity physical activity, *SB* sedentary time

All models are adjusted for age, sex, and highest household education

Statistically significant associations (*p* < 0.05) are highlighted in bold

Regression coefficients correspond to change in the log-ratio of the given behaviour compared to the other behaviours

## Discussion

Using a novel analytical method, this study examined the associations of sleep duration, sedentary time, LPA, and MVPA with BMI z-scores and waist circumference in a representative sample of Canadian children aged 3 to 4 years. The combined effects of these behaviours were significantly associated with BMI z-score, suggesting that the distribution of time between the movement behaviours across the whole day makes a difference. Descriptively, participants classified as overweight had lower MVPA and sleep duration, and higher LPA and sedentary time, relative to the overall sample. However, the time spent in sleep, sedentary behaviour, LPA, or MVPA relative to the other behaviours was not significantly associated with BMI z-scores in the linear regression models. Furthermore, no associations were observed with waist circumference.

The analyses used in this study of preschool-aged children are consistent with analyses conducted in school-aged children and youth (5–17 years) from cycles 1 to 3 of the CHMS [3]. Across both age groups, the composition of movement behaviours was associated with BMI z-score. However, in school-aged children and youth, associations were also seen with waist circumference. Furthermore, in school-aged children and youth, it was observed that relative to the time spent in other movement behaviours, MVPA and sleep were significantly positively associated, and LPA and sedentary behaviour were significantly negatively associated, with both BMI z-scores and waist circumference. Although the same direction of associations with BMI z-scores was observed in the linear regression models in the present sample, and although descriptively the compositional mean bar plots showed similar patterns, significant associations were not observed.

There are several potential reasons why the associations with BMI z-scores were less pronounced in the present sample of preschool-aged children compared to the previous sample of school-aged children and youth [3]. One noticeable difference between the two age groups is that preschool-aged children spent almost 10% more of their time sleeping within the 24-h period. In fact, half of the 24-h period was spent sleeping in this young age group. This is not a surprise, given it is recommended in Canada that preschool-aged children sleep 10–13 h/night [37], compared to 8–10 or 9–11 h/night for school-aged children and youth [1]. Nevertheless, the dominant amount of time spent sleeping among preschool-aged children would have reduced the time and potentially the variation of the other movement behaviours [38]. Furthermore, in response to the evidence that preschool-aged children’s movement is more sporadic and intermittent [39], cycle 3 of the CHMS changed accelerometer procedures to collect data in shorter 15-s epochs. Though efforts were made in this study to make data as comparable as possible between cycles 2 and 3 [28], the different accelerometer procedures between cycles may have introduced some error. Given that the preschool-aged sample was substantially smaller than the school-aged children and youth sample (552 versus 4169) [3], this additional error may have impacted the ability to detect statistical significance. Finally, movement behaviours may impact adiposity differently in preschool-aged children compared to older school-aged children and youth due to adiposity rebound [40, 41]. BMI typically declines after the child reaches 1 year and reaches its lowest point at the start of adiposity rebound, usually around the ages of 5 or 6 years [40, 42]. However, adiposity rebound can occur earlier or later, with early adiposity rebound associated with normal or low BMI prior to the start of the rebound and high BMI in adulthood [42]. There is some evidence to suggest that preschool-aged children who are highly active have later adiposity rebound [40, 41]. For example, an 8-year longitudinal study found that children defined as “high active” at 4 years old had higher BMIs than children defined as “low active” but the “high active” children had subsequent smaller gains in BMI throughout the study compared to the “low active” children [40]. Since adiposity rebound was not accounted for in this cross-sectional study, residual confounding may have impacted associations between the individual movement behaviours and BMI z-scores, relative to the time spent in the other movement behaviours.

Despite similar findings in the present sample and older samples for the association between the overall composition of movement behaviours and BMI z-score [3, 4], the lack of consistency in findings between the adiposity indicators BMI and waist circumference is unique to this sample of preschool-aged children. The measurement of waist circumference in addition to BMI is usually encouraged, given the link between abdominal obesity and cardiovascular disease in older age groups [43]. High correlations have previously been observed between BMI and waist circumference in children [44]. Therefore, it is unclear why findings were not consistent across adiposity indicators in this age group. It should be noted that age- and sex-specific z-scores based on international standards were calculated for BMI to take into account changes in height and weight that occur naturally through growth and development [30]. This appears particularly important in this age group, given the above discussions around adiposity rebound [40]. To the authors’ knowledge, similar international standards do not exist for waist circumference in this age group. Therefore, this study used the raw waist circumference measures in centimetres for all analyses. It is unknown whether this had any impact on the findings. Furthermore, there may be lower accuracy in measuring waist circumference, in comparison to heights and weights, in this young age group [45].

Given that this is the first study to examine the integrated associations between movement behaviours and adiposity in preschool-aged children, future research is needed to confirm these findings. While more direct measures of adiposity, such as dual X-ray absorptiometry, are logistically challenging in population-based studies such as the CHMS, future studies that utilize these more direct measures may provide further insight into these associations. Similarly, studies that utilize 24-h accelerometer protocols to obtain an objective measure of sleep may provide further insight. Furthermore, since movement behaviours may impact the timing of adiposity rebound in young children [40], future longitudinal studies are also warranted. It is important to note that adiposity is only one aspect of children’s overall health. It could be that other health indicators related to social, cognitive, or motor development may be more sensitive to the movement behaviour composition. However, these health indicators were not available in the CHMS in this age group. Therefore, these associations should also be explored in future research.

Although future research is warranted regarding integrated movement behaviours and health in preschool-aged children, the findings of this study still have some important implications. Specifically, the findings suggest that the overall composition of movement behaviours may be associated with BMI z-scores even in preschool-aged children. Therefore, in line with a recent WHO report on ending childhood obesity [16], targeting healthy movement behaviour patterns in the preschool age group appears to be a promising health promotion strategy. This is further supported by the evidence that movement behaviour patterns track from early childhood into later childhood [46], where associations have been observed with a variety of health indicators [3]. While the optimal composition of movement behaviours is still unknown in older age groups, new *Canadian 24-Hour Movement Guidelines for Children and Youth* recommend preserving adequate sleep, and replacing sedentary time and LPA with MVPA [1].

Strengths of this study include the representative sample of preschool-aged children, the compositional analyses, which took into account the co-dependent nature of the movement behaviour data, and the objective measures of sedentary time, LPA, and MVPA. However, as previously mentioned, different accelerometer procedures between cycles 2 and 3 may have introduced some error. Additionally, sleep was parental-reported and consequently the data needed to be normalized, which may also have introduced error. Furthermore, only sleep duration was captured and not sleep quality. Another limitation of the study is the cross-sectional data, which prevent a determination of the temporality between the composition of movement behaviours and adiposity indicators. Finally, findings may also have been influenced by residual confounding, as variables such as diet and timing of adiposity rebound were not controlled for in the analyses because they were not available in the CHMS.

## Conclusions

Research is growing on how we conceptualize and promote healthy 24-h integrated movement behaviour combinations. Compositional data analyses provide an appropriate method for analyzing the association between co-dependent movement behaviour data and health indicators. This study is the first to use this method when examining associations with adiposity indicators in preschool-aged children. The overall composition of movement behaviours was found to be associated with BMI z-scores but not waist circumference in this representative sample of 3 and 4 year olds. Future research is needed to determine the optimal composition of movement behaviours in this age group to inform health promotion efforts.

## Abbreviations

BMI: Body mass index
CHMS: Canadian Health Measures Survey
CPM: Counts per minute
LPA: Light-intensity physical activity
MVPA: Moderate- to vigorous-intensity physical activity
SB: Sedentary time
WC: Waist circumference
WHO: World Health Organization.
